# Case Report: Treatment of subthreshold bipolar disorder in pregnancy: a report on the effectiveness of the Zamar Protocol^®^ in three treatment-resistant depression cases

**DOI:** 10.3389/fpsyt.2026.1872443

**Published:** 2026-10-05

**Authors:** Antonios C. Zamar, Theodoros Koutsomitros, Georgios Mikellides, Angelos Halaris, George P. Chrousos

**Affiliations:** 1 The London Psychiatry Centre, London, United Kingdom; 2 Department of Cognitive Neuroscience, Faculty of Psychology and Neuroscience, Maastricht University, Maastricht, Netherlands; 3 Greek rTMS Clinic, Thessaloniki, Greece; 4 ® SynaΨe: European Medical Institute of Psychotherapy and Brain Stimulation, Thessaloniki, Greece; 5 Academy of Brain Stimulation, Maastricht, Netherlands; 6 Cyprus rTMS, Larnaca, Cyprus; 7 Department of Basic and Clinical Sciences, Medical School, University of Nicosia, Nicosia, Cyprus; 8 Department of Psychiatry and Behavioral Neurosciences, Stritch School of Medicine, Loyola University of Chicago, Chicago, IL, United States; 9 University Research Institute of Maternal and Child Health and Precision Medicine, Medical School, National and Kapodistrian University of Athens, and Hellenic Pasteur Institute, Athens, Greece; 10 UNESCO Chair on Adolescent Health Care, Athens, Greece

**Keywords:** FT3/FT4 ratio, HDT high dose levothyroxine, pregnancy, rTMS (repetitive transcranial magnetic stimulation), subthreshold bipolar disorder (SBD), treatment resistant depression (TRD), Zamar Protocol

## Abstract

**Introduction:**

Subthreshold bipolar disorder (SBD), also referred to as unspecified bipolar disorder, is frequently encountered in patients with treatment-resistant depression. It presents with depressive and subthreshold manic and mixed symptoms. Sustained remission with full functional recovery is the aim of treatment in mood disorders as residual symptoms may cause disability, while relapse may be associated with increased morbidity or mortality. To date, evidence for achieving sustained remission in SBD is absent, with no studies reporting sustained remission outcomes. The only available evidence to remission is at level 2 in a previous study by the author employing combined high-dose levothyroxine (HDT) and repetitive transcranial magnetic stimulation (rTMS). The management of SBD during pregnancy is particularly challenging due to the absence of evidence-based guidance.

**Method:**

We describe a retrospective case series of three women with longstanding treatment-resistant depressive presentations with a threat to life, later conceptualized within the bipolar spectrum. All patients had achieved sustained remission before pregnancy using a treatment approach combining HDT with rTMS (the Zamar Protocol), with or without low-dose quetiapine. Following shared decision making and counseling regarding the extremely limited evidence base, treatment was continued during pregnancy due to concerns about the riskof relapse and risk to life. All three patients maintained clinical stability during pregnancy, the postpartum period, and beyond. Across the three cases, five pregnancies occurred while patients remained on HDT-based treatment, with adjunctive rTMS used during selected periods. No maternal, obstetric, neonatal, or early developmental adverse outcomes were reported during the available follow-up. In one case, clinical deterioration followed a dose reduction and improved after re-escalation. The consistent biochemical pattern of elevated FT4 relative to FT3 (low FT3/FT4 ratio) previously reported was observed across cases, which differed from typical thyrotoxic profiles. Variants in the deiodinase-related genes and the thyroid hormone transport pathways were identified, in keeping with previously published literature.

**Conclusion:**

This small retrospective case series suggests that continuation of HDT-based treatment, with or without adjunctive rTMS, may be a feasible option in carefully preselected patients with pre-pregnancy blood and genetic biomarkers and pre-pregnancy clinical response when presenting with severe life-threatening bipolar spectrum disorder presenting as treatment-resistant depression. These findings established safety and efficacy at a low level of evidence (level 4 CEBM) and highlight the need for more robust prospective research. Careful discussion with the patient and spouse exploring the risk–benefit ratio and any evidence-based available acceptable alternatives, particularly when there exists a risk to life, is necessary.

## Introduction

Subthreshold bipolar disorder (SBD) or unspecified bipolar disorder, formerly known in the Diagnostic and Statistical Manual of Mental Disorders IV (DSM-IV) as bipolar disorder NOS (not otherwise specified), is a very common condition afflicting some 2.4% of the US population (2), while it is globally estimated to afflict 1.4% of the general population (2). The condition is as disabling as bipolar disorder I and II, particularly in the depressive phases being just as severe (3). The depressive and mixed phases predominate the clinical picture, with three-quarters presenting with anxiety disorders, the most common being panic attacks. Approximately 50% of patients with treatment-resistant depression suffer from SBD or major depressive disorder with subthreshold manic or hypomanic symptoms, i.e., “in the bipolar spectrum,” with up to 80% showing evidence of bipolarity (4).

There are no guidelines for the treatment of this condition, and it is known to be resistant to mood stabilizers even with added quetiapine, with failure to reach remission on such interventions (5). To our knowledge, evidence-based treatments capable of consistently achieving remission in SBD are absent, and there are no pharmacological studies specifically addressing the depressive component of the condition, except for one study combining mood stabilizers and quetiapine XR 300 mg, which failed to achieve remission (5). There are no effective remission-achieving treatments or any pharmacologic trials addressing the depressive component of this condition (5).

SBD is a rapidly cycling, treatment-resistant condition marked by brief periods of mixed, manic, or depressive symptoms—less than 7 days for mania, 4 days for hypomania, and 2 weeks for depression or mixed states—without clear episodes, but which can also occur in the context of bipolar I and II in between episode remissions (5). The Maudsley guidelines, 15th edition, 2025 (7), quoted only two studies covering SBD in the rapid cycling section: one study (8) with only one case in its mix and 45 cases in a protocol cohort study (6).

The Zamar Protocol^®^ has shown sustained remission in a cohort of 55 patients with rapid cycling bipolar disorder, of whom 45 were SBP, for an average duration of 2 years, with a large effect size of Cohen’s *D* of 2.6. The proposed mechanism of action of the treatment was highlighted in an article review by the first author (9). The protocol components are diagnostically guided repetitive transcranial magnetic stimulation (rTMS) based on differentiating the mixed from the depressive state, precision-guided high-dose levothyroxine (HDT) with parallel genetic tests with single nucleotide polymorphisms (SNPs) in deiodinase 1 (DiO1) and deiodinase 2 (DiO2) and/or the SLCO1C1 protein carrier of the thyroid hormones into the brain, and thyroid function test blood biomarkers with or without added quetiapine.

## Case reports

We report on three cases treated with the protocol during pregnancy, where all three patients made the conscious decision to get pregnant and proceed with HDT during pregnancy while being closely monitored. This is because a return to the illness after sustained remission was deemed an unacceptable risk to all three patients and their partners, especially in the postpartum period. All patients and their partners were counseled regarding HDT in pregnancy, highlighting the absence of data in the first patient and the presence of only one reported case in the second case and two cases in the third case. The patients and their partners decided to proceed with a pregnancy, while two (cases A and B) went on to have a second pregnancy after the first uneventful one.

### Case A

The patient was referred as an emergency in 2012 following a substantial deterioration of postnatal depression 18 months following delivery. Depression started within a week of birth, initially with suicidal thoughts with no intent or plans. However, at the time of the referral, the patient suffered suicidal thoughts, with plans to crash her car with her 18-month-old baby onto a wall at high speed due to very high levels of distress and despair. While depressed, she presented with severe anxiety, agitation, and symptoms of mania, including high levels of irritability and racing/crowded thoughts. There were previously no reported strictly manic or hypomanic episodes, although there were mixed episodes. A diagnosis of unspecified bipolar affective disorder (ICD-10 code: F31.9) was made. She was taken off citalopram—which is contraindicated according to the Food and Drug Administration (FDA) prescribing information of citalopram in mixed states and the contemporaneous past and current editions of the Maudsley prescribing guidelines in rapid cycling bipolar disorder—and started on quetiapine and rTMS. The response to treatment was partial with a maximum dose reached of 450 mg/day. There were significant side effects and residual symptoms, although suicidality abated. The patient complained of sedation until, upon reviewing the Maudsley guidelines, 12th edition, she opted to have HDT.

Her pretreatment scores were: Beck Depression Inventory II (BDI-II) = 33, Beck Anxiety Inventory (BAI) = 30, and Sheehan = 3.6. Her post-treatment scores were: BDI-II = 0 and BAI = 0. Sheehan was not completed, but was scored by implication given that the test scored the level of disabilities based on the symptoms, granted there were no symptoms at all and the score should be 0.

Levothyroxine was commenced, with the daily doses titrated by 50 μg/day weekly to 400 μg, with no side effects. She was euthyroid at the beginning of the treatment. She never reached full remission on quetiapine alone and was sedated, slowed down, and depressed. rTMS was commenced at 10 Hz left dorsolateral prefrontal cortex (DLPFC; F3, 5 cm anterior to the motor cortex) at 120% of the motor threshold, which improved her status. However, she remained slowed down with quetiapine. A joint decision to start rTMS 1 Hz right-side DLPFC (F4 in the 10–20 system, which is the mirror image of left F3, 5 cm anterior to the motor cortex) at 120% of the motor threshold with HDT was taken. The patient reached full remission on the HDT part of the protocol, with levothyroxine reaching 400 μg/day, and opted to reduce and discontinue quetiapine. She could not eliminate it altogether due to difficulty sleeping and has remained on 25 mg since. She remained well for approximately 2 years and then decided to get pregnant while on HDT. Thyroid function tests revealed thyroid-stimulating hormone (TSH) at less than 0.01 mIU/L, FT4 at 55.3 pmol/L, and FT3 at 8.8 pmol/L. The FT3/FT4 ratio was 0.16, which is substantially below the thyrotoxic range of 0.3 and above, and thyroiditis of 0.27. She experienced no side effects. Both endocrinology and obstetrics experts advised against HDT during pregnancy and asked her to reduce the dose to 150 μg/day. Following dose reduction, the patient deteriorated substantially despite having rTMS, and the HDT doses were increased until remission. Following multidisciplinary review including obstetrics and endocrinology and discussion of available alternatives, continuation of HDT was considered as the most appropriate option by the treating team and the patient. She gave birth to a boy, and both the mother and baby were apparently well. The child is now 12 years old, well adjusted, and with no behavioral or clinical issues. After 5 years, she decided to get pregnant again, but by then she was taking 250 μg/day. A common phenomenon observed in this patient population is that, with long remissions, patients start to develop minor hyperthyroidism side effects, necessitating dose reduction until they are symptom-free. The final dose of 250 μg/day suppressed TSH, but was associated with FT4 of 22 pmol/L and FT3 of 3.8 pmol/L, both within the normal range. The FT3/FT4 ratio was 0.17, again substantially lower than the thyrotoxic range despite concurrent TSH suppression. Although there was placenta previa in her pregnancy, it affected her husband’s stress levels more than the patient, with the obstetrics team duly concerned about both mother and baby. The patient stated that she was unstressed. The delivery was done by caesarean section, and both mother and baby were well. She described her last pregnancy as the best pregnancy she had despite the complication of placenta previa. The patient has been in full remission for 13 years after 10 years of continuous illness, exacerbated following the first pregnancy and inappropriately treated with antidepressants (sertraline and citalopram) prior to referral to our center. The second HDT child is now 6 years old and is mentally and physically well. The genetic profile of the child and, hence, also the placenta, showed heterozygous mutations in all thyroid pathway enzymes and carriers: DiO1 34>A, DiO2 rs12885300 Gly3Asp, DiO2 rs225014 Thr92Ala, and SLCO1C1 intron3C>T. Both babies were bottle-fed. This case failed to remit on rTMS combined with quetiapine, but remitted on the combination of HDT with the rTMS part of the protocol.

### Case B

Patient B presented with significant depressive and mixed symptoms, which continued for 5 years while she was on and off antidepressants. When assessed in 2009, she had 12 symptoms of hypomania from ICD-10 and scored 19 on the Hypomania Checklist-32 (HCL-32) questionnaire, giving a specificity of 89% (10), which indicates a 9 out of 10 chance of a bipolar illness. The bipolarity index score was 60, with a sensitivity of 91% and a specificity of 90% (11). The hypomanic phases lasted less than 4 days, making a diagnosis of unspecified (ICD-10 code: F31.9) or SBD most appropriate. The patient was treated with several drugs, including valproate, lithium, quetiapine, lamotrigine, olanzapine, and rTMS with quetiapine, olanzapine, and valproate, to no avail.

Response to treatment had been partial, and the side effects remained unacceptable. Her pretreatment scores were: BDI-II = 15, BAI = 4, and Sheehan = 7.8. This patient could not grasp the way to fill out the BDI-II and BAI. However, given that the level of impairment was very severe on the Sheehan Disability Scale, by implication the symptoms were severe, and so was the clinical picture conveyed. Her post-treatment scores were: BDI-II = 6, BAI = 4, and Sheehan = 0. The Sheehan Disability Scale in this case was the most important one given that mild depressive symptoms with flight of ideas should not indicate a mild disability.

Levothyroxine was administered alongside rTMS as per the protocol at 1 Hz right DLPFC (F4 in the 10–20 system, which is the mirror image of left F3, 5 cm anterior to the motor cortex) at 120% of the motor threshold, which brought about full remission at a final dose of 400 μg, with no side effects. The patient was euthyroid at the initiation of levothyroxine. Thyroid function tests showed a TSH of <0.01 mIU/L, FT4 of 43.4 pmol/L, and FT3 7.2 pmol/L. There was an elevated FT4/FT3 ratio, and the FT3/FT4 ratio of 0.16 was substantially below the thyrotoxic range.

The patient was newlywed just a year before the initiation of levothyroxine and decided to have a child while on HDT. She proceeded with rTMS 1 Hz right DLPFC during the initial stages of her pregnancy and had her first child. Both mother and baby were well, and the pregnancy, delivery, and puerperium were uneventful. The patient remained well for 2.5 years, and then decided to have a second child while on HDT. Again, the pregnancy, delivery, and puerperium were uneventful, and the second child is now 2 years old. Both babies were bottle-fed.

She continued with 400 μg of levothyroxine, again with no side effects. The HDT-based treatment approach was associated with sustained long-term remission in this patient after several previous interventions provided only partial or transient benefit and led to consecutive uneventful pregnancies and delivery of healthy babies, with the mother remaining well. Case B showed heterozygous mutations with SNPs in both DiO1 rs2235544 34C>A and DiO2 rs225014 Thr92Ala genes. This case failed to remit on mood stabilizers and second-generation antipsychotics (SGAs). Remission was achieved on HDT combined with the rTMS part of the protocol.

### Case C

Patient C presented following an inpatient admission and after at least three episodes of severe depressive symptoms with what transpired to be flight of ideas of negative thoughts together with agitation and suicidal ideation and intent, which led to several weeks of inpatient stay. Several antidepressants were attempted, including sertraline, mirtazapine, vortioxetine, and trazodone, as well as cognitive behavioral therapy (CBT). The condition deteriorated on all interventions. A diagnosis of a mixed affective state without episodes of hypomania or mania was made, namely, depression with flight of ideas. Her pretreatment scores were: BDI-II = 37, BAI = 51, Sheehan = 8.3, and Impact of Event Scale (IES) = 21. Her post-treatment scores: were BDI-II = 6, BAI = 4, Sheehan = 0, and IES = 3.

As there had been no reported manic or hypomanic episodes, a diagnosis of unspecified bipolar disorder ICD-10 code: F31.9/ICD-11code: A6Z6 was made. rTMS at 1 Hz right-side DLPFC (F4 in the 10–20 system, which is the mirror image of left F3, 5 cm anterior to the motor cortex) at 120% of the motor threshold for 1 week, which was then switched to high-frequency (HF) 20 Hz right DLPFC (F4 in the 10–20 EEG system, 5 cm anterior to the motor cortex) at 120% of the motor threshold, was started together with quetiapine up to 300 mg, which was not tolerated. She was then switched to olanzapine 15 mg. After 8 weeks, there was no benefit but sedation and weight gain. The patient then came under the care of the author when the HDT part of the protocol was started and reached a final dose of 1,150 μg, with 50 μg weekly increments. The patient then went into full remission.

Patients may show some minor side effects, such as feeling hot or sweating, following remission, with the dose of levothyroxine then reduced and maintained at the dose at which there are no side effects and the patient remains emotionally stable. We postulate that this occurs because of the “switching off” of the relevant gene expression after remission. The patient required 600 μg/day. The couple decided, without notifying the treating team, to have a baby and informed the team of the pregnancy after the event. The risk of HDT withdrawal-related relapse and the use of alternative approaches were discussed with the couple, with cases A and B mentioned to the patient as the only existing data. The paucity of relevant data was emphasized. Nonetheless, the patient and her husband chose to continue HDT, encouraged by the fact that cases A and B had very positive outcomes previously. She continued with 600 μg/day throughout the entire pregnancy. Both pregnancy and delivery proceeded uneventfully, apart from 1 day of baby blues. The baby is now 18 months old and apparently well.

There was no relapse and no side effects, and the baby had normal thyroid function and normal developmental milestones. The thyroid function tests were TSH = 1.77 mIU/L (range, 0.27–4.2), FT4 = 14.8 pmol/L (range, 12–22), and FT3 = 3.5 pmol/L (range, 3.1–6.8), with an FT3/FT4 ratio of 0.23 pre-Zamar Protocol^®^, and during pregnancy were TSH = 0.01 mIU/L, FT4 = 32.1 pmol/L, and FT3 = 5.3 (normal), with an FT3/FT4 ratio of 0.16. The lag of conversion of FT4 to FT3 is evident, as is the case in this group of patients.

Genetic testing showed heterozygous mutations in DiO1 rs2235544 34C>A and DiO2 Thr92Ala rs225014 and homozygous mutations in DiO2 Gly3Asp rs12885300 and the SLCO1C1 intron3C>T protein transporter of the thyroid hormone in the brain tissue. The baby was exclusively breast-fed, with no complications. This case failed to remit to rTMS combined with SGA, but remitted on the HDT combined with the rTMS part of the protocol.

## Outcomes

All three cases suffered from severe, treatment-resistant, and life-threatening symptoms. None of the cases had a history of 7 days of mania or 4 days of hypomania, ruling out specified bipolar disorder, namely, bipolar I and II, respectively. However, all presented with alternating mixed and depressive states, hence the diagnosis of unspecified bipolar disorder (ICD-10 code: F31.9/ICD-11 code: 6A6Z). Case A was planning to crash her car with her child at high speed onto a wall and was referred as an emergency. Case B had tried multiple psychotropic medications from different classes to no avail, while she declined electroconvulsive therapy. Case C attempted suicide and was admitted to hospital.

All three patients were diagnosed following a positive screen on the HCL-32 questionnaire and having shown and reported alternating periods of less than 2 weeks of depression with flight of ideas and/or agitation with brief and less than 4-day duration of elevated mood or simple depression, which were confirmed by history, collateral history, and clinical examination.

SBD is an extremely common condition, but which very few psychiatrists are aware of, with neither guidelines for its management nor treatment or clinical trials (5) for drug treatment. One study in young people with subthreshold bipolar symptoms comparing CBT to unstructured group meetings found no difference between groups in terms of improvement rather than remission over a 6-month period. Unstructured group meetings are cheaper and easier to arrange than CBT, effectively negating the need or the use of CBT in this condition, despite the conclusion of the authors (12).

All three patients were counseled regarding the lack or the paucity of data on HDT during pregnancy, and all had rTMS combined with HDT before pregnancy. Case B had only HDT only in the second pregnancy, while cases A and C had added rTMS combined with HDT due to ongoing stressors during pregnancy. None had quetiapine or any other psychotropics, except for case B at 25 mg to aid sleep.

None of the patients or their babies suffered any apparent negative sequelae from HDT. There are many studies, including that of the first author’s, showing the absence of side effects in the bipolar disorder population (7, 13). However, to date, there have been no reports of HDT during pregnancy. The outcomes for the babies relied on the standard developmental milestones of newborns and children in the UK and on parental reports. The developmental milestones are standardized through the National Health Service (NHS) and the Early Years Foundation Stage (EYFS), which include physical, communication, and social skills from 0 to 5 years.

## Discussion

All cases were diagnosed by first screening with the HCL-32 screening questionnaire, followed by diagnosis using the screening questions for mania from the Composite International Diagnostic Interview (CIDI) version 3.0 previously used in the WHO Merikangas study (3), where the presence of one screening question of the CIDI version 3.0, such as flight of ideas together with failure to meet the threshold of 4 days of hypomania, accurately diagnosed SBD. This is an accepted WHO diagnostic test for the condition. Case B was re-diagnosed with the Bipolarity Index, which has a very high sensitivity and specificity in diagnosing bipolar disorder (12, 14). These are level 1 research-grade diagnostic tests where the clinical diagnosis is regarded as unacceptably poor (15).

Thyroxine (T4) is the inactive prohormone that is activated by conversion to T3 both in the periphery, centrally, and in specific organs. The enzymatic pathways involved in the activation of T4 converting it to T3 are through DiO1 in the liver and kidney and DiO2 in the brain, heart, and fetoplacental unit. A third enzyme, deiodinase 3 (DIO3), which is abundant in many tissues including the placenta and breast, deactivates T3 by reconverting it to T2 and back to T4, as well as reverse T3 (rT3), which in turn competes with T3 at the intracellular receptor level (16, 17).

The remaining question was how the babies did not suffer any detriments on HDT in these three cases. Theoretically, this is possible (level 5 CEBM 2011), as DIO3 inactivates T4 and T3 to rT3 and T2, respectively, and is highly expressed in the placenta, decidua, fetal endothelium, and many fetal tissues, protecting against maternal thyroid hormone excess (18). In fact, a study highlighted that the placental DIO3 activity is orders of magnitude higher than that of DiO2 (roughly 100- to 400-fold in the human placenta across gestation), ensuring that even substantial maternal T4 or T3 elevations are, in essence, buffered before reaching the fetus (19).

In thyrotoxicosis, a high T3/T4 ratio (meaning T3 elevation is marked relative to T4) is evident, as opposed to that in HDT treatment of bipolar disorder, where T4 is substantially higher than T3, with a substantial increase in rT3 (6). When the maternal free T4 or free T3 may be high–normal or abnormally elevated, the placental DiO3 converts much of this excess T4/T3 to inactive metabolites, preventing fetal thyrotoxicosis (20). Because HDT in bipolar disorder shows the exact opposite in terms of the FT3/FT4 ratio to thyrotoxicosis, the placental physiology in this population should be considered differently from that of thyrotoxic patients.

Typical blood biomarkers of a high FT4/FT3 (low FT3/FT4) ratio (7), coupled with the SNP genes coding for DiO1 and DiO2, are present compared with Graves’ disease and thyroiditis, which show the opposite, i.e., a high FT3/FT4 (low FT4/FT3) ratio. The diagnostic cutoff in thyrotoxicosis for the FT3/FT4 ratio is 0.28–0.30 when concentrations are expressed in picomoles per liter (21). A mean cutoff point of the FT3/FT4 ratio of 0.22 at day 10, which then dropped to 0.2 at remission, measured and maintained through the final test over a median observation period of 2 years, was previously reported (7). We speculate that epigenetic changes in the genes protecting the fetus from excessive maternal free T4 and T3 by neutralizing them through deiodination might have occurred, explaining the lack of adversity at delivery or later. As DiO1 and DiO2 are the enzymes responsible for the conversion of the inactive prohormone T4 to the active hormone T3—and DiO3 deactivates excess T4/T3 during HDT treatment—the levels of T3 are not as elevated, being limited by DiO1 in the maternal circulating blood, and then, as a second layer of protection, DiO3 deactivates any elevated iodothyronine at the fetoplacental unit. This potentially explains the mother’s wellbeing during pregnancy. It is also worth highlighting that the enzyme SNPs observed can and do occur in the general population, as highlighted previously (22). However, the issue is whether these enzymes are expressed and the phenotype, i.e., the blood test FT4/FT3, demonstrates a change in the FT3/FT4 ratio. Furthermore, as a third protection level, the placental DiO2 SNPs may cause a decrease in the activation of T4 to T3, allowing less T3 to pass through the placenta, hence protecting the fetus.

In all babies, the thyroid function tests were normal 5 days after delivery, indicating no adverse effect of HDT on the babies’ thyroid physiology.

Regarding breastfeeding, both DiO2 and DiO3 are present in the breast tissue (23), potentially exerting effects similar to those in the placenta in the presence of the above SNPs in DiO2 (20).

In effect, it could be argued that pregnancy and lactation are protected from HDT in this population of patients. However, more data are required before making such an assertion.

Taken together, during pregnancy, the absence of symptoms of hyperthyroidism in this population, together with an FT3/FT4 ratio below 0.28, indicates that there is no thyrotoxicosis, which serves as a reassuring blood biomarker. The presence of SNPs in the thyroid activating pathway also helps confirm the level of protection, but with the blood biomarkers highlighting gene expression. Close follow-up during pregnancy by psychiatric and endocrinology experts is warranted, simply because of the dilution of circulating thyroid levels and because the patient’s mental state can deteriorate, as happened in case B when the dose was reduced to physiologic levels by the endocrinologist. In this case, a threat to life to both mother and baby had to be considered, which was the case previously when the mother was contemplating crashing her car onto a wall.

To our knowledge, this observational case series represents the first published report describing the continuation of HDT-based treatment in patients with severe bipolar spectrum illness during pregnancy and lactation. The lengths of remission are 13 years for case A, 9 years for case B, and 2 years for case C after decades of progressive and unresponsive illnesses. Of note is that the sample size is too small to support definitive conclusions regarding safety.

These cases illustrate the clinical dilemma posed by severe treatment-resistant bipolar spectrum presentations during pregnancy, particularly when prior deterioration has been marked and the relapse risk is high. In such circumstances, continuation of a previously effective treatment may be considered following individualized multidisciplinary assessment and shared decision making. Our observations suggest that HDT-based treatment, with adjunctive rTMS in selected cases, did not appear to be associated with any short- and long-term maternal or infant harm in this small series for up to 12 years post-delivery (level 4 CEBM). All three cases failed to achieve satisfactory remission with rTMS and/or quetiapine; hence, we employed the rTMS protocol combined with thyroid hormones and/or quetiapine (24). With regard to efficacy, this is supported by a level 2, grade B recommendation, representing the highest available level of evidence in SBD. The scores relate to remission, which was achieved pre-pregnancy as all three cases were well when they became pregnant. Remission was maintained throughout pregnancy and beyond, except in case A when a dose reduction was attempted at the request of the endocrinologist, which only resulted in significant deterioration. The treatment decisions were individualized and were based on prior clinical response, illness severity, relapse risk, multidisciplinary assessment, and shared decision making with patients and their families. The efficacy of the protocol has been established with a very large effect size (2.6) in a cohort of 55 patients over an average follow-up period of 2 years (CEBM level 2) and was effective in these three cases for many years. The biochemical pattern observed across the three cases is similar to the ones reported in a cohort of 199 patients by the author (22) and justifies further investigation. Prospective registries and multicenter observational studies are needed.

With regard to the potential long-term risks due to exposure to HDT, including cardiovascular and bone mineral density (BMD), many long-term studies (13) and two randomized controlled trials (RCTs) (13, 25) described in systematic reviews (26, 27) have shown that such risks do not occur in men and non-pregnant females, even in postmenopausal women, with some studies showing mixed results in the latter population (28). The three cases were under medical supervision with periodic electrocardiograms (ECGs) and BMDs, as well as reviews by psychiatry, cardiology, and endocrinology, and still continue to be under cardiology supervision. This is not because of the theoretical cardiovascular risk of HDT but because of the proven, beyond any doubt, cardiovascular risk (29) and the 30-year cardiovascular morbidity and mortality risks associated with bipolar disorder, which starts from age 18, effectively when the disease starts (30). The presence of SNPs is not necessarily enough to reflect gene expression (https://www.psychiatrycentre.co.uk/wp-content/uploads/2023/05/US-patent-11638831-certificate.pdf), and epigenetic environmental factors, including inflammation, infections, stress, injury, and alcohol and drug use, may play important roles in gene expression and the phenotype presentation. Part of the phenotype is the blood biomarker showing the subnormal FT3/FT4 ratio, suggesting potential effects of the SNPs on gene expression.

## Conclusion

SBD is a clinically impairing and likely under-recognized condition for which treatment guidance is absent, particularly during pregnancy. In this retrospective series, the continuation of HDT-based treatment, with adjunctive rTMS in some cases, was associated with sustained maternal psychiatric stability and no observed adverse outcomes in the mothers or infants during the available follow-up. However, given the very small sample size, the retrospective design, the lack of controls, and potential selection bias, these observations should be interpreted with caution. The biochemical pattern of high FT4 relative to FT3 has been demonstrated in a previous study at level 2b CEBM and warrants further studies in pregnancy. HDT has been shown to be safe and effective over many decades in men and non-pregnant women. Prospective, multidisciplinary studies are needed before any conclusions can be drawn regarding safety or generalizability, although major ethical issues will remain as obstacles for controlled studies in pregnancy. The protocol as it stands is administered in a single specialized center, and specialist training and expertise is required for its implementation.

## Data Availability

The raw data supporting the conclusions of this article will be made available by the authors, without undue reservation.
